# Teaching emergency situations during a psychiatry residency programme using a blended learning approach: a pilot study

**DOI:** 10.1186/s12909-021-02887-2

**Published:** 2021-09-06

**Authors:** Juliette Salles, Philippe Birmes, Laurent Schmitt, Bruno Bastiani, Maria Soto, Stéphanie Lafont-Rapnouil, Anjali Mathur, Emmanuelle Bougon, Christophe Arbus, Antoine Yrondi

**Affiliations:** 1 Service de Psychiatrie et Psychologie, Department of Psychiatry and Psychology, CHU Toulouse (Toulouse University Hospital Centre), F-31000 Toulouse, France; 2 INSERM U1043, Infinity, Université Paul Sabatier (Paul Sabatier University), Toulouse, France; 3 Toulouse NeuroImaging Centre, Université de Toulouse (Toulouse University), Inserm, UPS, Toulouse, France; 4 Service de Psychiatrie et de Psychologie Médicale, Department of Psychiatry and Medical Psychology, Centre Expert Dépression Résistante FondaMental (Expert Centre for Treatment-Resistant Fundamental Depression), CHU Toulouse, Hôpital Purpan, Toulouse, France; 5 Institut Toulousain de Simulation en Santé (ITSIMS), Toulouse Institute for Health Stimulation, Toulouse, France; 6 Alzheimer’s Disease Research and Clinical Centre, INSERM U 1027, Toulouse University Hospital, Gerontopôle, France; 7 Pole de Psychiatrie (Psychiatric Emergency Department, Psychiatric Unit), Urgences Psychiatrique, CHU Toulouse, Hôpital Purpan, Toulouse, France; 8 Service de Psychiatrie et de Psychologie Médicale, Department of Psychiatry and Medical Psychology, Centre Thérapie Brève, Short Treatment Centre, CHU Toulouse, Hôpital Purpan, Toulouse, France; 9 Service de Psychiatrie et de Psychologie Médicale, Centre Expert Dépression Résistante FondaMental, CHU Toulouse, Hôpital Purpan, ToNIC, Toulouse NeuroImaging Centre, Université de Toulouse, Inserm, UPS, Toulouse, France

## Abstract

**Background:**

Emergency psychiatry is an essential component in the training of psychiatry residents who are required to make patient-centred orientation decisions. This training calls for specific knowledge as well as skills and attitudes requiring experience. Kolb introduced a theory on experiential learning which suggested that effective learners should have four types of abilities: concrete experience, reflective observation, abstract conceptualisation and active experimentation.

We aimed to evaluate a resident training programme that we designed for use in an emergency psychiatry setting based on the experimental learning theory.

**Methods:**

We designed a four-step training programme for all first-year psychiatry residents: (i) theoretical teaching of psychiatric emergency knowledge, (ii) concrete experience of ability teaching involving an initial simulation session based on three scenarios corresponding to clinical situations frequently encountered in emergency psychiatry (suicidal crisis, hypomania and depressive episodes), (iii) reflective observation and abstract conceptualisation teaching based on videos and clinical interview commentary by a senior psychiatrist for the same three scenarios, (iv) active experimentation teaching during a second simulation session based on the same three frequently encountered clinical situations but with different scenarios.

Training-related knowledge acquisition was assessed after the second simulation session based on a multiple-choice quiz (MCQ), short-answer questions and a script concordance test (SCT). The satisfaction questionnaire was assessed after the resident had completed his/her initial session in order to evaluate the relevance of teaching in clinical practice. The descriptive analyses were described using the mean (+/- standard deviation). The comparative analyses were conducted with the Wilcoxon or Student’s t tests depending on data distribution.

**Results:**

The residents’ mean MCQ and short-answer question scores and SCT were 7.25/10 (SD = 1.2) 8.33/10 (SD = 1.4), 77.5/100 (SD = 15.8), respectively. The satisfaction questionnaire revealed that 67 % of residents found the teaching consistent.

**Conclusion:**

We designed a blended learning programme that associated, classical theoretical learning to acquire the basic concepts, a learning with simulation training to experiment the clinical situations and a video support to improve learning of interview skills and memory recall. The residents indicate that this training was adequate to prepare them to be on duty. However, despite this encouraging point, this program needs further studies to attest of its efficiency.

**Supplementary Information:**

The online version contains supplementary material available at 10.1186/s12909-021-02887-2.

## Background

Emergency training is essential for mental health care as it represents the gateway to the mental health care system for many patients [1]. A psychiatric emergency is defined by the American Psychiatric Association as “an acute disturbance in thought, behaviour, mood, or social relationship, which requires immediate intervention as defined by the patient, family, or social unit”. Thus, patients referred to psychiatric emergency units could present severe symptoms such as suicidal behaviour or delusion and hallucination [2]. Those clinical situations need prompt intervention to prevent imminent danger [3]. Moreover, clinical practice in psychiatric emergency situations requires professionals to make the right diagnosis within a short period of time [4, 5]. Consequently, training in specific skills is required in emergency psychiatry.

Moreover, these skills should be taught as part of a dedicated programme including a psychiatry resident module. Indeed, residents often start their residency programmes in psychiatric emergency units and are also placed on call. Teaching emergency psychiatry to residents therefore poses a major challenge and involves the learning of practical skills. The latter cannot be learned through simple observation and specific teaching methods involving experiential learning are required for this purpose.

Kolb introduced an experiential learning theory which is recognised as an efficient learning model. It suggests that effective learners should have four types of abilities: concrete experience, reflective observation, abstract conceptualisation and active experimentation. According to Kolb, learning requires individuals to initially detect knowledge. A construction phase should then take place to consolidate the learning process [6]. Considering those elements, we designed a training that followed the experiential learning cycle of Kolb. To achieved this, we used a blended learning defined as a combination of various teaching approaches [7]. Blended learning for postgraduate health professionals is relatively new, as is the focus on the social interactive component of online learning, but it seems efficient and well accepted by students [8].

For concrete experience until now, learning has mainly taken place in emergency units with real patients. However, this approach is no more consistent with recent pedagogic developments that recommend starting clinical practice without real patients [3, 9–11], particularly on ethical grounds [12]. In order to firstly experiment clinical situation without real patients, the simulations training is now in widespread use for professional [13]. This is also the case in psychiatry indeed simulation training is increasing [14–16], and specifically for teaching emergency situations [17, 18]. Simulation is characterized as follows by McGaphie [19]: “Simulation procedures for evaluation and teaching have several common characteristics: (i) Trainees see cues and consequences very much like those in the real environment; (ii) Trainees can be placed in complex situations; (iii) Trainees act as they would in the real environment; and (iv) the fidelity (exactness of duplication) of a simulation is never completely isomorphic with the ‘real situation.” Despite the limitation pointed by the last point, this tool could be considered as interesting for concrete experience.

In addition to the scenario playing, the simulation training includes a debriefing time that could be considered as a reflective observation since it is a method used to scrutinize a learner’s own assumptions and professional work practices.

In order to improve the quality of the learning of psychiatric interview it is also important for the learner to be able to abstract and conceptualize the knowledge. That is to say be able to make sense of what has happened in the interview, in order to interpret the events and the relationships between the actions. At this stage the learner needs to compare what he has done or observed, to reflect it upon and to link it with theoretical knowledge. To help this process it could be helpful for the learner to refer on others interviews including those from senior colleagues. In addition, it will be valuable to associate those interviews with a pedagogical description of what happen during the interview to serve as models of comprehension. For this reason, we think that it could be interesting to make video support of psychiatric interview (in the same situations as simulation) and to associate them with commentaries. Moreover, video cases focusing on psychiatric patient case management have been suggested to enhance students’ insight before meeting patients in real-life scenarios [20, 21]. The effects of using teaching formats such as video cases have been widely studied in various training programmes focusing on suicide risk [22], assessment of ethical sensitivity [23], attitudes towards psychotherapeutic techniques [24] and the perceived dangers associated with psychiatric patients [25]. In addition, video cases using simulated psychiatric patients in interactive teaching formats have been suggested to stimulate patient-centred communication when conducting the diagnostic interview [26, 27] and appear to be better than text-based material during training programmes [28]. Moreover, the video approach is a powerful teaching and learning tool because the incorporation of images into the educational process increases learning retention and visual images offer several advantages over verbal communication [29, 30]. Furthermore, videos provide more information in a given space and time, simplify complex concepts, clarify pieces of abstract language-based concepts, and demonstrate concepts/subjects that are in motion and/or relate to one another [31]. Consequently, we found that the use of videos could be of interest in relation to abstract conceptualisation.

Finally, the active experimentation could be represented by a second session of simulation and then by the confrontation with the first real clinical situation such as the first on-call activities.

**Fig. 1:**
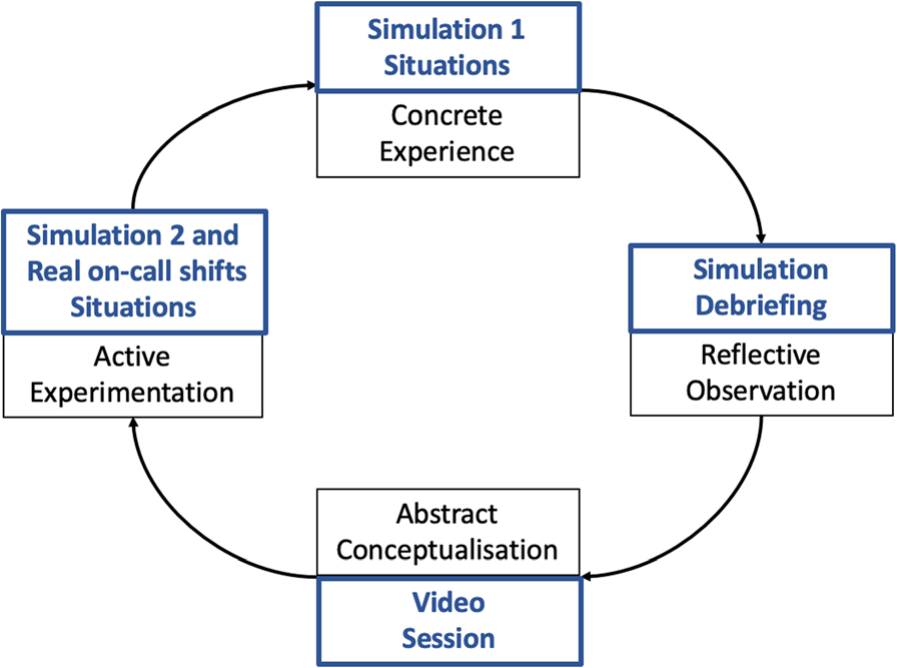
Description of the learning strategy based on Kolb’s Experiential Learning Cycle. The schema described what pedagogic tool was chosen for each stage of learning

The Fig. 1 summarized the teaching approach.

We then assessed training-related knowledge acquisition. The theoretical learning was evaluated by short-answer questions and multiple-choice quizzes. The clinical reasoning process was evaluated using the script concordance test [32]. For SCT, we also compared the residents’ responses to the senior practitioner’s answer in order to evaluate the decision-making process based on decisions taken by experienced psychiatrists in the same situations. The relevance for real practice was assessed using a satisfaction questionnaire.

Our aim is to set up a new learning strategy including theoretical classes, simulation, video, MCQ and SCT for the psychiatric residents to teach them the conduct of clinical interviews and manage care decisions in emergency situations. We described the complete procedure of this teaching and the teaching outcomes in this article.

## Methods

### Setting

We conducted this teaching session at the School of Medicine, Paul Sabatier University, Toulouse (France), at the *Institut Toulousain de Simulation en Santé* (ITSIMS), which replicates a fully equipped emergency room. The workstation is equipped with ceiling cameras that recorded the simulations.

### Participants

First-year psychiatry residents participated in a mandatory simulation activity to address frequent emergency situations. Twenty-three residents participated in the teaching programme they were divided into two groups: one with 12 residents and one with 11 residents. Sixty percent of whom were women and forty percent men. The mean age of the students was 25.5 years (SD = 3.8).

### Description of the teaching programme

**Fig. 2:**
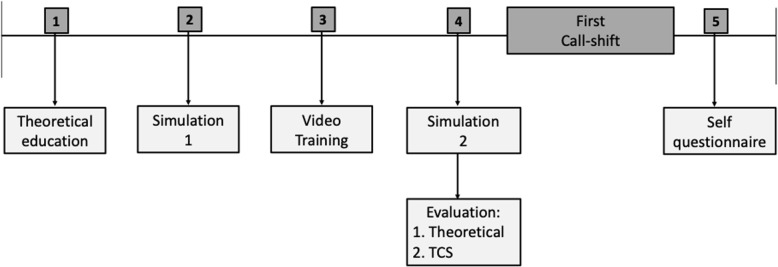
Flowchart

The teaching component was divided into four sessions spread over four consecutive weeks (before starting the emergency activity) and ended with an assessment (Fig. 2). Firstly, all residents took courses focusing on emergency situations in psychiatry. These courses were based on data taken from a national framework of competencies that are required in psychiatry. The aim of these courses is to introduce the most commonly encountered emergency situations and to provide the specific skills to manage these types of situations. These courses were delivered via lectures and online courses. Two simulation sessions (sessions 1 and 3) were then carried out and one video training session (session 2). The patient was played by an actor previously trained in psychiatric disorders. The supervisors (JS, MS, LS, PB, CA, AY) designed simulation scenarios to reproduce real-life clinical situations occurring in the psychiatric emergency setting: major depressive episodes, suicidal crises and hypomania.

The objectives of the teaching programme were to learn and use psychiatric assessment techniques to address the question of suicide risk and to learn and use psychiatric knowledge to assess major depressive episodes, suicidal crises and hypomania episodes.

The first session was a simulation session. This session started with the “pre-event” (called pre-brief) preparation of the residents where rules and expectations (objectives) were explained to the participants. At this point, the environment was described as non-threatening, confidential, and “psychologically safe”. After the pre-brief, one resident faced the first clinical situation for 20 min (on a voluntary basis). Residents had to interview the patient in order to look for symptoms. Depending on these symptoms, residents had to provide appropriate care. The standardised patient was instructed to become progressively more ambivalent about this care in order for residents to deal with a frequently encountered emergency room situation.

Other residents were in the debriefing room watching the situation as it was filmed. At the end of this first clinical scenario, a debriefing session was held with the whole group and the supervisors (JS, BB, AY) (45 min). We used the PEARLS blended debriefing approach in our own simulation. Debriefing comprised four phases: a reactions phase, a description phase, an analysis phase and a summary phase [33]. The second resident then faced the second clinical scenario and the third resident the last clinical scenario. Each clinical situation was followed by a debriefing session. The six residents who performed the first simulation were assessed during debriefing, based on a check list of required skills. We assessed how the resident introduced themselves [their attitude (presentation/eye contact etc.)]. Secondly, we assessed how they investigated (i) clinical symptoms to diagnose a disorder and assess the level of suicidal risk, (ii) comorbidities such as alcohol misuse/abuse/dependence, and (iii) other medical conditions. Thirdly, we assessed how the resident helped identify potential resources accessible to the patient (e.g. family/friend support networks, resources to find employment or housing etc.). Finally, we assessed how the resident concluded the medical consultation (focusing on the care recommendations).

The second session was a video session. Three senior psychiatrists working in the emergency department faced the same clinical situations (played by the same actor). These three clinical situations were recorded. The video was then edited using Camtasia^®^ software to select the relevant points in the video to illustrate the difference in approach between the resident and the senior psychiatrist. The videos were also edited to last 10 to 15 min in order to keep the resident focused. The teaching team provided clinical information verbally and answered the residents’ questions for the purpose of an interactive approach and practical experience transmission. A member of the ITSIMS teaching team also participated in this session in order to provide knowledge about cognitive bias that occurs in immersive situations.

We specifically chose to show the senior psychiatrist video after the first simulation session to prevent the student from seeing the clinical situation and trying to prepare for it. We also wanted residents to apply their newly acquired understanding, gained from theoretical training, to the clinical context. We presumed that showing the senior psychiatrist video before the first simulation would influence behaviour by giving students grounds to imitate the senior assessor. The objective of the simulation exercise was for students to experiment with their own abilities and overcome their own difficulties. Furthermore, we wanted to use the senior practitioner’s video as part of the debrief.

The third session was a simulation session with a different scenario from the first session but with reference to the same three clinical situations. A different group of residents now faced the “patient”. The six residents who performed simulation were assessed during the debriefing session following the same procedure as in the first session.

To sum up, we divided our teaching into four components: firstly, residents were given a presentation focusing on the skills to be acquired in emergency practice in accordance with current international recommendations. This was followed by the first simulation session. A training video on improving the skill set was then shown. Finally, residents took part in the second simulation session which focused on the same disorder but with a slightly different scenario. We chose specific media to teach the different skills to be acquired (regular classroom setting and video training) as well as the two simulations (two different scenarios concerning the same disorder) in order for residents to develop their own skills whilst complying with the principles of repetition through practice/teaching.

### Assessment

All of the residents were assessed after the theoretical learning phase at the end of the second simulation session.

Theoretical learning was assessed by short-answer questions and multiple-choice quizzes (Supplementary file 1). In simulation, for higher-order cognitive assessment (knowledge, application, and synthesis of knowledge), context-based MCQ, and short answer questions are appropriate [34].

Experiential learning was evaluated using the SCT tools described by Kazour et al. [35]. SCTs have been developed to assess clinical reasoning in uncertain situations deemed to reflect the uncertainty of clinical situations in practice [36]. The SCT principle is to test the concordance between the students’ clinical reasoning processes and those of reference experts [37].

The SCTs are composed of short vignettes describing clinical situations in psychiatry. Here, we developed four different SCTs from the three clinical situations using videos edited by the senior psychiatrist. These clinical situations reflect real cases with different management options. Five items follow each vignette. Each item is associated with a treatment option, followed by new relevant information, the impact of which on the initial option was assessed according to a five-point Likert scale, − 2 (very unlikely), − 1 (unlikely), 0 (neither likely nor unlikely), + 1(more likely), + 2 (likely). The Likert scale was used to establish whether the new information made the proposed treatment option more or less useful. We chose this evaluation method as it allowed the student to develop clinical relevance in line with clinical practice. In the SCT, residents were asked about the decisions they made for patient care (outpatient or inpatient care) taking new information into consideration.

A final session was organised to discuss corrections regarding the MCQ, short-answer questions and SCT with the residents.

Finally, we gave a self-questionnaire to the residents 1 month after starting their on-call shifts to inquire whether the teaching programme was consistent with reality (Supplementary file 2). This questionnaire interrogated if the type of situations encountered in on-call shifts were similar to the scenario proposed in the simulation, the resident answered yes or no. It also interrogated if the scenario reflected the real clinical situations, if the proposed teaching was a consistent training for the on-call shits, if the theoretical knowledges acquired during the teaching were useful for the on-call shits. Finally, the resident had also to give a satisfaction score on ten points (0 for a very unsatisfying teaching, 10 for a very satisfying teaching).

### Statistical analysis

**Fig. 3:**
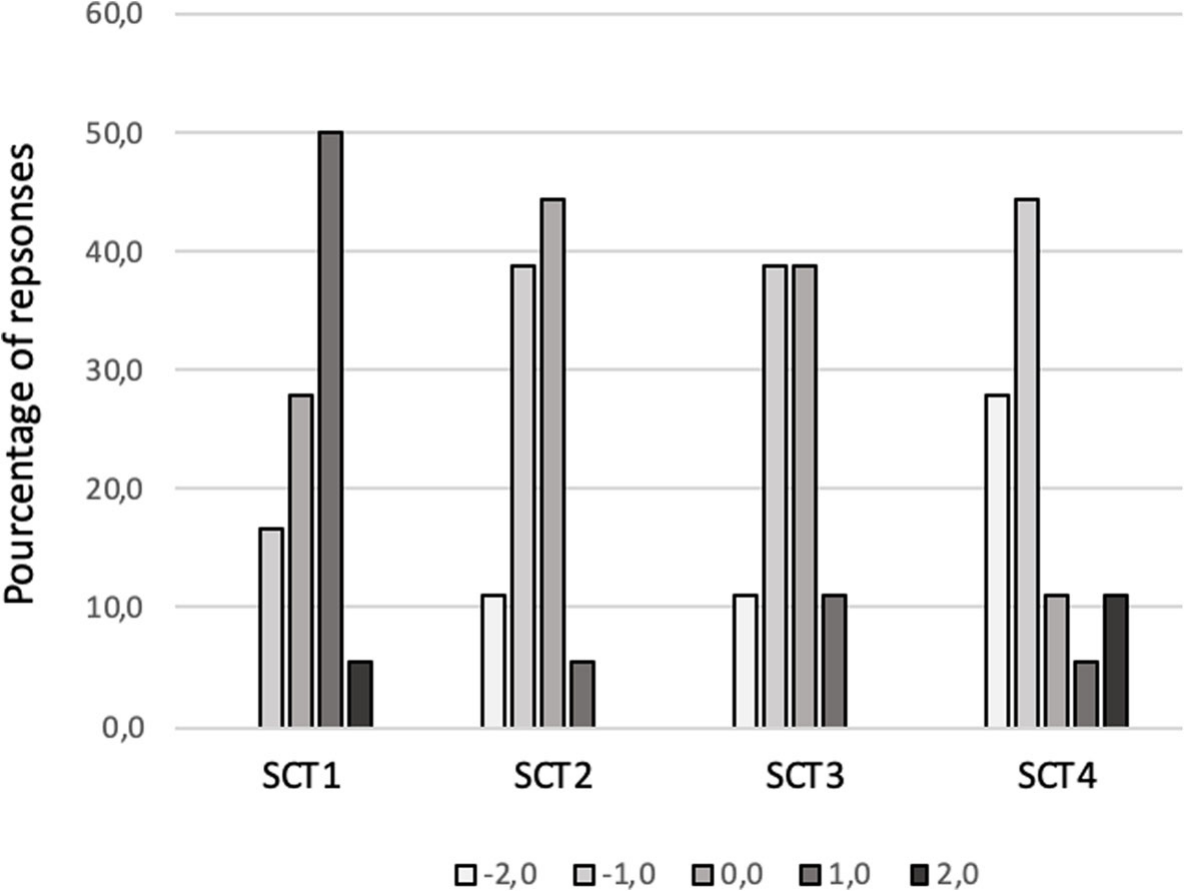
Description of script concordance test (SCT) answers by senior psychiatrists. The number above the bar graph indicates the percentage response for each proposal

We conducted a descriptive analysis of our data in which quantitative variables are described using the mean (+/- standard deviation) and/or median (+/- interquartile range) according to their distribution. With regard to SCT, the responses from the senior panel were used to score the assessment based on standard methods [36, 38]. For each item, the credit refers to the number of panel members who chose that answer, divided by the modal value for the question. If, for a given question, 15 of our 18-strong panel chose “-2,“ two chose “-1” and one chose “0”, the credit for “-2” is 0.83 (15/18), that for “-1” is 0.11 (2/18) and the credit for “0” is 0.05 (1/18). For the non-chosen options, “+1” and “+2,“ the credit is 0. With this method, all questions have the same maximum (1) and minimum (0) value. The Fig. 3 present the results of the SCT scoring in the senior group that permitted the quotation of the scores for the resident. The score obtained for each SCT are added and divided by the number of the SCT to obtain a global mean score for all the SCT. We then multiplied this mean score by 100 to express it in a percentage format.

To analyse the resident decision-making process, we focused on the impact of new information on the initial care direction. Indeed, the SCT focused on the care direction and initially proposed outpatient care in two cases and inpatient care in the other two instances. We considered that the decision-making process differed between senior psychiatrist and resident when the mean score between the groups was conflicting (one group revised its direction while the other group did not). We focused on the care direction decision and two SCTs to analyse the decision-making process.

For the satisfaction questionnaire we performed descriptive analysis regarding the number of responses by item, the scores are presented in percentage.

The statistical analyses were performed using Excel software and R Studio version 1.1.453 – © 2009–2018.

## Results

For the MCQ evaluation, the mean score was 7.25/10 (SD = 1.2).

For the short-answer questions, the mean score was 8.33/10 (SD = 1.4).

Eighteen senior psychiatrists completed the SCT. Forty-four percent of them have practised psychiatry for more than 10 years since their residency, 39 % have practised psychiatry for 2-10 years since their residency, and 17 % have practised psychiatry for less than 1 year since their residency. Sixty-two percent were women and thirty-eight percent were men. All of the senior psychiatrists currently work on-call in the psychiatric emergency ward.

For the residents the mean SCT score was 77.5/100 (SD = 15.8). The minimal score was 2.1 and the maximal was 4. The mean SCT1 score was 69.4/100 (SD = 38.8), 70.8/100 (SD = 27.8) for SCT2, 82.8/100 (SD = 25.5) for SCT3 and 87.2/100 (SD = 23.5) for SCT4.

Considering the decision-making process, we found that decisions were conflicting for two of the SCT. As regards SCT1 for which the initial proposal was outpatient care, the senior psychiatrist confirmed this decision after new information had come to light while the resident revised the decision. For SCT3 in which the initial proposition was patient care, the senior psychiatrist revised the decision to outpatient care while the resident confirmed the inpatient approach. A difference between the two groups was noted for SCT2 (initial inpatient care) and SCT4 (initial outpatient care).

In the self-questionnaire responses, 89 % of the residents indicated that during their on call-shifts they encountered the same type of clinical situation that those taught in the simulation sessions. Twenty-seven percent of residents found the teaching to be very consistent with the reality of being on-call, 67 % found it consistent, and 6 % not very consistent. Twenty-five percent of the residents deemed the scenario to be very close to clinical reality and seventy-five percent estimated the scenario to be close to the real situation experienced on-call. Forty percent found knowledge teaching very useful for their on-call practice, and sixty percen found it useful. The mean satisfaction score was 8.3 (SD = 1.04).

## Discussion

To our knowledge, this is the first residents’ programme using simulation and video teaching in emergency psychiatry. This programme corresponds to current trends in medical teaching to avoid initial practice with a real patient. It also responds to the residents’ request to be better prepared for their first on-call shift. It was devised in order to integrate the conventional teaching of theoretical knowledge with simulation practice, and to provide concrete examples of psychiatric assessments made by senior psychiatrists.

This approach was designed to meet all four stages of Kolb’s learning cycles, [6] with (1) Concrete Experience, (2) Reflective Observation, (3) Abstract Conceptualisation, and (4) Active Experimentation. It combined concrete experience and engaged learners through reflective observation (with the simulation session debriefings), through abstract conceptualisation (comparing a resident assessment to a senior psychiatrist assessment during a screening session with commentary) and through active experimentation (i.e. application in simulation 2 and future practice in the emergency unit). In order to aid the transfer of theoretical knowledge to practical application, we asked the senior psychiatrist, who teaches the theoretical course, to perform the expert video. This demonstrated how the concepts taught are implemented in practice.

We found that theoretical learning was acquired by the resident and noted satisfactory scores in the MCQ short answers test and SCT. However, we were not able to conclude that this teaching improves the learning versus a more traditional one.

Apart from the scoring, a valuable aspect of the SCT was also to place the resident in a virtual decision-making situation where new information had to be integrated [39]. The care strategy often has to be modified in practice as a result of additional information and the SCTs were designed to reflect clinical situations in which students have to interpret data to make decisions [32, 40]. Moreover, we thought that the decision to integrate SCT evaluation by a psychiatrist using interview videos, as in other medical disciplines (videos of endoscopies or neurology symptoms) [41], facilitates immersion in a clinical situation and better prepares residents for clinical reasoning when on-call. This evaluation showed that the resident made a different decision compared to the senior psychiatrist in terms of revising inpatient care or confirming outpatient care. Indeed, in this case, the resident’s decision was to implement inpatient care. However, residents are less likely to consider patient discharge than senior psychiatrists.

As previously described [42], we found that the simulation debriefing gave the resident an active role in asking questions about the knowledge taught in previous courses. Moreover, the video provided concrete examples for formulation questions. We noted that the student learned from this approach and used it in the second simulation session. Video material for teaching psychiatry already exists, especially online, as provided by the American Psychological Association (APA) [43]. Nevertheless, we think that video presentations outlining similar clinical situations to those taught in the simulation session may facilitate the transfer of knowledge.

Residents appeared to be satisfied in terms of the value gained from this type of programme. They found it to be close or very close to clinical reality. We emphasise this last point because it confirms that the residents could use the knowledge acquired through simulations to perform their clinical assessment in real situations, as was previously noted in simulation learning [43]. This is relevant since a theory-practice gap has been identified in the teaching of medicine and nursing [44].

There were some limitations to this study: (1) As this was a pilot study, it was conducted with a relatively small sample within a single residency programme. (2) There was no control group using standard teaching to compare the real efficacy of this programme. (3) Earlier studies used methods to test the reliability and validity of the SCT [45, 46], but we did not conduct any such analysis in our study.

4) Considering there was no comparison with an identical pre-test, it is impossible to conclude that the residents scored well on the test as a direct result of the training as opposed to other learning experiences (e.g. medical school experience). Further studies should focus on assessing residents with the same tools prior to training. 5) The residents appreciated the learning. However, we have not asked them about the individual training components and the teaching tools used may vary in terms of appeal. Moreover, we did not assess the contribution of each tool to the learning strategy in order to prove that the blended approach generated a real improvement versus teaching based purely on theoretical learning or teaching by simulation learning or teaching by video. 6) Qualitative expression with technical methods and qualitative content analysis could help to deepen the experience. We plan to use qualitative assessment in subsequent studies.

Finally, some residents commented that the clinical situations should be more difficult in order to be more representative of complex patients. This point is open to discussion as we deliberately chose simple scenarios. Our decision was based on two aspects. Firstly, we knew that there were differences between residents in terms of the level of experience because they were not all trained in the same psychiatric unit (some of them were in a paediatric psychiatry unit, for example). Hence, we opted for simple situations so as not to discourage residents with less clinical experience. We also deemed it inappropriate to risk putting the residents under additional pressure during the simulation, as simulations are stressful exercises in which each resident is observed by the group. Secondly, our teaching approach for complex situations was to refer the scenario to the senior psychiatrist before making a clinical decision. We therefore thought it could be misleading to choose complex situations as examples and preferred to teach simple cases to deliver basic messages. We believe that, conversely, the limits outlined by the students could be a strength of our programme.

Despite these study limitations, we consider our training approach to be original as only a few programmes use the four stages of Kolb’s learning cycle [38, 47]. These steps seem crucial to process key learning outcomes. Thus, we set up this training programme just weeks before the residents commenced real-life practice in the emergency unit.

## Conclusions

We designed a blended learning approach that could be valuable in a resident training programme in psychiatry. The originality of this programme is to associate different pedagogic tools corresponding to the stages of the cycle of Kolb. We found it to be feasible and adequate for the psychiatric residents. However, despite those points, this program needs further studies including studies with specific skill-based assessments to attest of its efficiency.

## Supplementary Information

**Additional file 1: Supplementary file 1.** Theoretical learning was assessed by multiple-choice quizzes and short-answer questions. Theoretical learning assessment

**Additional file 2: Supplementary file 2.** Self-questionnaire. Teaching programme assessment

## Data Availability

The data sets used and/or analysed during this study are available from the corresponding author on request.

## Abbreviations

ITSIMS: *Institut Toulousain de Simulation en Santé*
MCQ: Multiple choice quizzes
MDD: Major depressive disorder
SCT: Script concordance test
