# Supplementary material for: Teaching emergency situations during a psychiatry residency programme using a blended learning approach: a pilot study
Source: BMC Med Educ. 2021 Sep 6;21:473. doi: 10.1186/s12909-021-02887-2 (PMC8419928; doi:10.1186/s12909-021-02887-2)
Supplement: Supplementary file 1 — Additional file 1: Supplementary file 1. Theoretical learning was assessed by multiple-choice quizzes and short-answer questions. Theoretical learning assessment [file 12909_2021_2887_MOESM1_ESM.docx]

Suicide: Which propositions are true?

A: 10% of the population declares that they have attempted Suicide

B: 30% of the population claim to have attempted Suicide?

C: Of those who have had a suicide attempt, 40% will reoffend, half of them within the year

D: Among people who have had a suicide attempt, 20% will relapse, half of them within the year

E: 10-15% of people doing a suicide attempt will die by suicide

Answers: A ; C ; E

Which of the following items are risk factors for suicide?

A: Psychiatric disorders

B: Family history of suicide attempts

C: Impulsivity

D: Social isolation

E: Non-psychiatric disorder

Answers: A; B; C ; D

What should we evaluate when a patient has suicidal ideation?

A: Intensity

B: Frequency

C: Duration

D: Cause

E: Deterrent factors

Answers: A; B; C; D; E

1 / Name the three major etiological frameworks to be evoked during an agitation (give two examples for each)

2 / Name 5 points of de-escalation

3 / What treatment (class) should we use during an agitation in the context of alcohol withdrawal

4 / What should an observation in psychiatric emergencies include?

5 / What treatment can we administer to a patient with an acute stressful state?

6 / A patient with a high risk of suicide need a hospitalization However, he refuses this hospitalization and forbids me to contact a family member. What are the possible options?
