# Supplementary material for: Teaching emergency situations during a psychiatry residency programme using a blended learning approach: a pilot study
Source: BMC Med Educ. 2021 Sep 6;21:473. doi: 10.1186/s12909-021-02887-2 (PMC8419928; doi:10.1186/s12909-021-02887-2)
Supplement: Supplementary file 2 — Additional file 2: Supplementary file 2. Self-questionnaire. Teaching programme assessment [file 12909_2021_2887_MOESM2_ESM.docx]

**Survey**

1) Did you work in emergency unit?

2) If yes, did you encounter any situations discussed in simulation?

3) If yes, which situation (s)?

4) If yes, you have found the simulation cases:

A. Very far from the real situation

B. Far from the real situation

C. Close to the real situation

D. Very close to the real situation

5) In any case, you found the course:

A. Not at all suitable for preparing guards

B. Not suitable for preparing guards

C. Adapted to the preparation of guards

D. Very suitable for the preparation of guards

6) In emergency unit, the knowledge taught to you were:

A. Not at all useful

B. Not useful

C. Useful

D. Very useful

7) Either way, you found the video training session:

A. Not at all useful

B. Not useful

C. Useful

D. Very useful

8) Did you conduct an interview during the simulation?

9) On a scale of 0 (dissatisfied) to 10 (very satisfied), how do you assess this teaching?

10) Suggestions in order to improve this teaching.
